# Discrimination of normal oral mucosa from oral cancer by mass spectrometry imaging of proteins and lipids

**DOI:** 10.1007/s10735-018-9802-3

**Published:** 2018-11-03

**Authors:** Katarzyna Bednarczyk, Marta Gawin, Mykola Chekan, Agata Kurczyk, Grzegorz Mrukwa, Monika Pietrowska, Joanna Polanska, Piotr Widlak

**Affiliations:** 1 Faculty of Automation, Electronics and Computer Science, Silesian University of Technology, ul. Akademicka 16, 44-100 Gliwice, Poland; 2 Center for Translational Research and Molecular Biology of Cancer, Maria Sklodowska-Curie Institute – Oncology Center Gliwice Branch, ul. Wybrzeze Armii Krajowej 15, 44-101 Gliwice, Poland

**Keywords:** Head and neck cancer, Mass spectrometry, Molecular classification, Molecular imaging, Lipidomics, Proteomics

## Abstract

Identification of biomarkers for molecular classification of cancer and for differentiation between cancerous and normal epithelium remains a vital issue in the field of head and neck cancer. Here we aimed to compare the ability of proteome and lipidome components to discriminate oral cancer from normal mucosa. Tissue specimens including squamous cell cancer and normal epithelium were analyzed by MALDI mass spectrometry imaging. Two molecular domains of tissue components were imaged in serial sections—peptides (resulting from trypsin-processed proteins) and lipids (primarily zwitterionic phospholipids), then regions of interest corresponding to cancer and normal epithelium were compared. Heterogeneity of cancer regions was higher than the heterogeneity of normal epithelium, and the distribution of peptide components was more heterogeneous than the distribution of lipid components. Moreover, there were more peptide components than lipid components that showed significantly different abundance between cancer and normal epithelium (median of the Cohen’s effect was 0.49 and 0.31 in case of peptide and lipid components, respectively). Multicomponent cancer classifier was tested (vs. normal epithelium) using tissue specimens from three patients and then validated with a tissue specimen from the fourth patient. Peptide-based signature and lipid-based signature allowed cancer classification with a weighted accuracy of 0.85 and 0.69, respectively. Nevertheless, both classifiers had very high precision (0.98 and 0.94, respectively). We concluded that though molecular differences between cancerous and normal mucosa were higher in the proteome domain than in the analyzed lipidome subdomain, imaging of lipidome components also enabled discrimination of oral cancer and normal epithelium. Therefore, both cancer proteome and lipidome are promising sources of biomarkers of oral malignancies.

**Electronic supplementary material:**

The online version of this article (10.1007/s10735-018-9802-3) contains supplementary material, which is available to authorized users.

## Introduction

Cancer located in the head and neck region is the sixth most common cancer worldwide, accounting for above 4% of cancer cases overall (Jemal et al. 2011). In excess of 95%, these are head and neck squamous cell carcinomas (HNSCC), a term which refers to cancers derived from stratified squamous epithelium lining mucosa of the upper aerodigestive tract, including the mouth, pharynx, sino-nasal tract and larynx. In most cases, the etiology of HNSCC is clearly understood and involves exposure to tobacco and alcohol, which are key factors, with an increasing proportion of oropharynx cancers associated with human papillomavirus. Globally, HNSCC accounts for about 600,000 new cases and 300,000 deaths per annum. However, HNSCC remains a relatively under-researched cancer with continued poor prognosis and significant treatment challenges. Despite recent improvements in treatment, HNSCC prognosis remains unfavorable, with less than 50% of patients remaining alive after 5 years. HNSCC is a heterogeneous disease and cases with similar pathologic features can differ in clinical outcome. The analysis of data available in The Cancer Genome Atlas revealed the existence of genetically distinct subgroups of HNSCC that could be further separated based on transcriptional profiling (Leemans et al. 2018). The generally accepted molecular biomarkers to guide management of HNSCC patient are still missing, hence therapeutic decisions are solely based on tumor localization and traditional staging. Therefore, there is a constant and urgent need to identify and validate biomarkers for molecular classification and stratification of HNSCC (Corvò 2007; Bose et al. 2013). HNSCC is managed with surgery and/or chemoradiotherapy. Since surgery is the primary treatment in most HNSCC cases, uncompleted resection of primary tumor could be a reason for treatment failure due to local recurrence. The histopathological examination is used to determine the adequacy of surgical resection of the tumor. However, this analysis could miss out sub-microscopic and/or pre-cancerous spots. Thus, determination of molecular factors discriminating between cancerous and normal mucosa for proper delineation of tumor area remains another critical issue in the field of molecular diagnostics of HNSCC (de Carvalho et al. 2012).

Mass spectrometry imaging (MSI) is a powerful approach allowing for a unique combination of molecular and morphological information. In this technique series of pixels across the surface of tissues are scanned with the use of a mass spectrometer, which generates multiplex space-correlated mass spectra. Mass profiles of different molecular species (proteins, lipids, metabolites, etc.) revealed by MSI can be spatially resolved and annotated with morphological and histological structures. This feature makes MSI not only complementary but also superior to the classical pathology (rev. in Caldwell and Caprioli 2005; Cornett et al. 2007; McDonnell and Heeren 2007; Seeley and Caprioli 2011). MSI based on MALDI ionization (MALDI–MSI) remains the most popular technique in biomedical research, which proved its role as a powerful tool in clinical proteomics, with obvious applicability in biomarker research and molecular tissue classification. This approach was used for molecular characterization of different types of cancer, including lung, breast, prostate, gastric, larynx cancers and brain tumors (rev. in: Aichler and Walch 2015; Schwamborn and Caprioli 2010; Schöne et al. 2013). The particular advantage of MSI in cancer research is an allocation of molecular profiles to specific types of cells and tissues. Moreover, MSI can be used in studies aimed at the interface between tumor and normal tissue (e.g., tumor niche and molecular margins), the contribution of heterotypic material in solid tumors and intra-tumor heterogeneity (Caldwell et al. 2006; Oppenheimer et al. 2010; Kang et al. 2010; Jones et al. 2013; Alexandrov et al. 2013; Balluff et al. 2015).

MALDI–MSI was recently used to characterize proteome profile of oral cancers. This approach allowed to detect differences between normal mucosa and squamous cell cancer, and to reveal different tumor sub-regions putatively corresponding to the cancerous epithelium and cancer-modified stroma (Widlak et al. 2016). Lipidome is another molecular component of cells involved in proliferation and differentiation, immunity and inflammation. Hence, metabolism of lipids and their specific cellular distribution is related to growth and invasion of cancer (Santos and Schulze 2012; Beloribi-Djefaflia et al. 2016). MALDI–MSI showed that lipid profiles could discriminate cancer and stroma cells in oral tumors (Uchiyama et al. 2014). Here we matched molecular pictures derived from imaging of two domains of molecular components of HNSCC — proteins, and lipids, aimed at direct comparison of their ability to discriminate cancerous and normal oral mucosa and to estimate their potential usefulness as a source of novel hypothetical biomarkers.

## Materials and methods

### Clinical material

Tissue material was collected from four patients (three males and one female; 36–59 years) who underwent surgery due to head and neck squamous cell carcinoma located in tongue: Case_1 — cancer stage T4N2M0, Case_2 — stage T4N2bM0, Case_3 — stage T1N0M0, Case_4 — stage T2N0M0. In all cases, surgery was the primary treatment (no neo-adjuvant chemo- or radiotherapy was involved). The fresh post-operative material was evaluated by an experienced pathologist, then tissue specimens were immediately frozen and stored at − 80 °C. Each tissue sample was cut into 10 µm serial sections using a cryostat, then H&E stained and examined by a pathologist either without or post-MSI analysis. The study was approved by the appropriate Ethical Committee (Maria Skłodowska-Curie Institute, approval number KB/430-17/13 from 12/03/2013), and performed in accordance with European, national and institutional guidelines.

### Preparation of samples for MALDI–MSI

Cryo-cut 10 µm tissue sections were placed onto ITO-coated conductive slides and dried under vacuum for 40 min. For peptide imaging slides were washed twice in 70% ethanol and once in 100% ethanol (each wash for 1 min), followed by 1 h drying, then coated with a solution of trypsin (Promega, 20 µg in 200 µL of 50 mM NH_4_HCO_3_) using an automatic spraying device (ImagePrep; Bruker Daltonik, Bremen) and incubated in a humid chamber for 18 h at 37 °C to perform tryptic digestion of proteins. No additional sample pre-treatment was performed for lipid imaging. To obtain optical images, the slides were scanned using a flatbed scanner before matrix deposition. Both for peptide and lipid imaging a solution of 2,5-dihydroxybenzoic acid (DHB; 30 mg/mL in 50% methanol and 0.2% TFA) was deposited onto the surface of tissues with the use of ImagePrep device (using the Bruker’s standard matrix coating program with doubled phase 5).

### MALDI–MSI analysis

Tissue sections were imaged using a MALDI-TOF/TOF ultrafleXtreme mass spectrometer (Bruker Daltonik, Bremen) equipped with a smartbeam II™ laser operating at 1 kHz repetition rate. Ions were accelerated at 25 kV with PIE time of 100 ns. Spectra were acquired in positive reflectron mode in the 800–4000 mass range for peptide imaging or in the 300–1200 mass range for lipid imaging; external calibration with Bruker’s Peptide Calibration Standard or cesium triiodide clusters was performed for peptide and lipid imaging, respectively. A raster width of 100 µm was applied, 400 spectra were collected from each ablation point. Compass 1.4 for FLEX series (Bruker Daltonik, Bremen) was employed for spectra acquisition, processing and creation of primary images. After analysis slides were rinsed twice in 100% ethanol to remove the matrix, stained with hematoxylin and eosin, and scanned for co-registration with MALDI images using FlexImaging 4.1 software (Bruker Daltonik, Bremen). Original files with spectra were converted into .txt files using FlexAnalysis 1.4 software (Bruker Daltonik, Bremen) for further analyses.

### Spectra processing

The basic preprocessing steps included: spectrum resampling, adaptive baseline correction (Bednarczyk et al. 2017), identification of the outlying spectra (as those with too big or too small TIC) with the use of Bruffaerts’ criterion for extremely skewed distributions (Bruffaerts et al. 2014), spectra alignment to the average spectrum based on Fast Fourier Transformation (Wong et al. 2005), and TIC normalization. Gaussian mixture model (GMM) approach (Polanski et al. 2015) was used for the average spectrum modeling and peak detection. GMM components of high variance and/or low amplitude were filtered out reducing the data dimensionality. The GMM components modeling the right-skewed spectrum peaks were identified and merged with the left-neighboring major component. The final set of GMM components was termed molecular components hereafter, which represents peptide and lipid species detected by MS. The abundance of the particular molecular component was estimated by convolution of GMM components and every spectrum.

### Comparative analysis

The coefficient of variation was used as the measure of molecular components’ dispersion. The Lilliefors test was performed to verify the hypotheses on the normality of molecular component abundance distribution across the tissue, while F test was applied to check on variance homogeneity. The significance of differences in abundance of each molecular component between cancer ROIs and normal epithelium ROIs was calculated by Mann Whitney U test with Benjamini–Hochberg correction for multiple testing. The Cohen’s effect was estimated based on trimmed mean and pooled Winsorized standard deviation (Wilcox and Tian 2011). Pairwise similarity index was calculated based on the mean spectra of cancer and epithelium ROIs for a different number of top peptide and lipid components (Frank et al. 2008).

### Molecular image segmentation

A spectra clustering procedure, named divisive iK-means algorithm (Mrukwa et al. 2016) was used to determine sub-regions in tissue preparations. The algorithm was used for each tissue specimen separately and for all samples combined. The splitting was stopped if the cluster size was less than the a priori assumed threshold value (1% of the original tissue size) and/or the intra-cluster distance distribution was unimodal.

### Spectra classification

The logistic regression technique was applied to the problem of spectra classification between cancer and (normal) epithelium ROIs. The classifier was trained on the sample set composed of Case_1, Case_2, and Case_3; using the multiple random validation scenario (50 iterations) with the split: 50% for training and 50% for testing set. Due to a significant imbalance of ROIs size (overrepresentation of cancer regions), the random downsampling procedure was applied to correct after it. Bayesian Information Criterion was used for model selection. The molecular signatures found in each of the random validation iterations were used to rank the features. The feature scoring performed for every iteration was calculated based on the model accuracy obtained for the testing set, the presence of the feature in the particular signature, and, finally, the importance of the feature in the classifier signature (the first feature chosen during the classifier training was treated as the most important, while the last one — as the least important). The obtained scoring allowed to construct the rank of features that helped in the selection of the final classifier signature. The knee rule supported by the analysis of the classifier stability were used to select the features. The obtained classifier was then validated on an independent tissue sample (Case_4). Pairwise Pearson’s correlation coefficients between the features from the final classifier signatures were calculated and only those statistically significant and of high effect size were reported.

### LC–MALDI MS/MS analysis and identification of molecular components

Each tissue sample analyzed by MALDI MSI was used for protein identification using shotgun LC–MS/MS approach. Protein lysates were prepared according to Protocol 1 and subjected to tryptic digestion according to a modified version of a combination of FASP with Stage-Tip fractionation (Wiśniewski et al. 2009) as described in Protocol 2, both given in the Supplementary Material. Tryptic peptides were then separated using an EASY-nLC nano-liquid chromatograph (Proxeon) coupled with PROTEINEER fc II fraction collector (Bruker) and analyzed using ultrafleXtreme MALDI–TOF/TOF mass spectrometer. A detailed description of instrumental settings of the LC–MALDI–MS/MS system is given in the Supplementary Material (Protocol 3). Registered MS/MS spectra were exported to ProteinScape 3.1 software (Bruker Daltonik) and analyzed using Mascot Server 2.5.1 (Matrix science, London, UK); for details see Protocol 4 (Supplementary Material).

The hypothetical identity of molecular components was established via assignment of m/z values of peptide and lipid species detected by MALDI MSI to masses of species with a known identity. M/z values of peptide components were attributed to measured masses of peptides identified in LC–MALDI–MS/MS experiment described above. The assignment was performed allowing ± 0.05% mass tolerance. M/z values of lipid components were annotated using SimLipid (PREMIER Biosoft) software. Search parameters were set according to MALDI-MSI experiment conditions, specifying positive ion mode with protonated ion [M+H]^+^ and two cation adducts: [M+Na]^+^ and [M+K]^+^. Targeted lipids were determined for three main categories: glycerolipids, glycerophospholipids, and sphingolipids. The acceptable error tolerance for m/z values was set to 0.5 Da. In the case of more than one matched lipid per MSI molecular component, the most probable hit (with the lowest mass delta) was attributed.

## Results

Four tongue tissue specimens containing squamous cell cancer and normal epithelium (i.e., oral mucosa) were analyzed by MALDI–MSI. Serial sections of each specimen were processed to allow imaging of molecular components corresponding to peptides (in principle resulting from proteins digested with trypsin; spectra registered in the 800–4000 mass range) and lipids (mostly “neutral” zwitterionic phospholipids; spectra registered in the 300–1200 mass range). There were 2435 spectral components identified in the peptide domain and 2108 spectral components identified in the lipid domain, which represented different molecular species and their isotope envelops. There were 5000–12,000 space-oriented spectra registered for each tissue specimen. Three tissue specimens (Case_1, Case_2, and Case_3) were used as a training set to establish molecular differences between cancerous and normal epithelium, while the fourth specimen (Case_4) was used only for validation of the obtained cancer classifier.

**Fig. 1:**
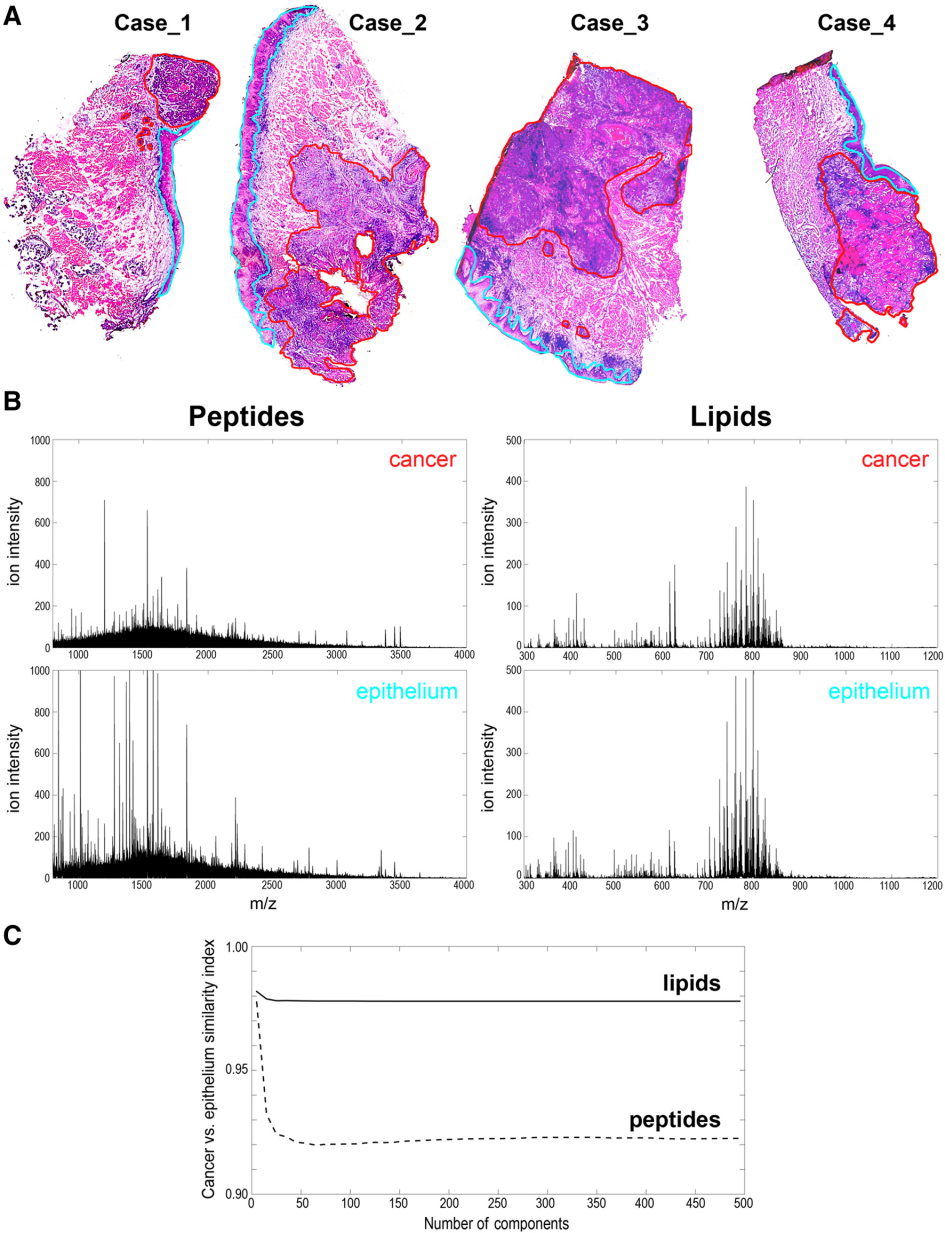
Analysis of oral cancers by MALDI–MSI. **a** Tissue specimens stained with H&E to visualize basic histology; cancer and epithelium ROIs were delineated with red and blue lines, respectively (shown are samples imaged for peptides). **b** Average peptide and lipid spectra computed for cancer and epithelium ROIs from Cases 1–3. **c** Similarity index between cancer and epithelium ROIs from Cases 1–3. (Color figure online)

Tissue regions corresponding to cancer and (normal) epithelium were delineated by an expert (i.e., an experienced pathologist) after molecular image registration (Fig. 1a), and spectra from these two types of ROIs (regions of interest) were exported for further analyses; average spectra registered for cancer ROIs and epithelium ROIs are presented in Fig. 1b. In general, average “lipid” spectra from cancer and epithelium ROIs were more similar than the corresponding “peptide” spectra. This observation was illustrated in Fig. 1c, where a similarity index between pairwise analyzed cancer versus epithelium ROIs was estimated in the peptide and lipid domains.

**Fig. 2:**
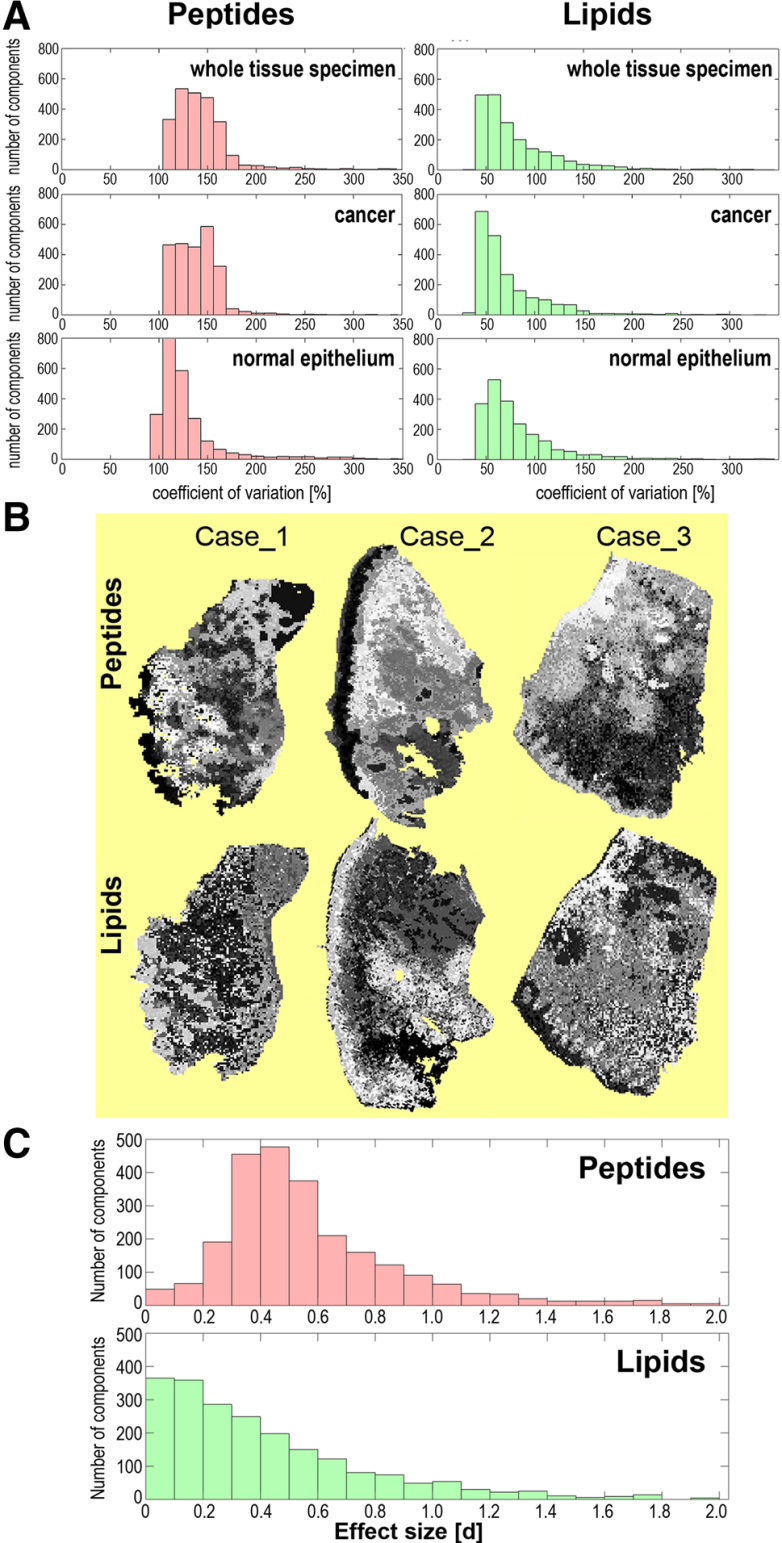
Variability of abundances of molecular components detected by MALDI-MSI. **a** Uniformity of components estimated by their coefficient of variation in whole tissue specimens or cancer and epithelium ROIs. **b** Results of unsupervised image segmentation; each tissue specimen was processed individually. **c** The significance of differences between cancer and epithelium ROIs. Combined cancer ROI and epithelium ROI of three samples from the training set were analyzed

The variability/uniformity of intensities (abundances) of peptide and lipid components through all spectra (i.e., measurement points) from the training set was first compared. Histograms in Fig. 2a represent the distribution of coefficient of variation calculated for each component; more variable components were characterized by a higher coefficient of variation. In general, variation in abundance of peptides was higher than variation in abundance of lipids. This is noteworthy, however, that variation in abundance of both peptides and lipids was higher within cancer than within normal epithelium. To further compare uniformity of peptide and lipid components unsupervised segmentation of molecular images was performed (Fig. 2b), and the size of the resulting clusters was compared. In general, there were similar numbers of clusters and average sizes of clusters found in both peptide and lipid domains (Table 1). However, the size of the largest clusters was usually 2–4 higher in the domain of lipids, which also reflected higher overall homogeneity of lipid images.

**Table 1:** Results of unsupervised image segmentation

Molecular domain	Case_1		Case_2		Case_3	
	Peptides	Lipids	Peptides	Lipids	Peptides	Lipids
Number of clusters	1251	1535	962	1479	1719	1633
Average size of a cluster (%)	0.08	0.07	0.10	0.07	0.06	0.06
Size of the largest cluster (%)	6.88	10.45	4.68	19.93	3.10	10.83

Size of an average and the largest cluster is presented as a percentage of the whole specimen area

Considering a higher variation of peptide components than lipid components we hypothesized that discrimination between different tissue regions could be easier in the peptide domain. To verify this hypothesis we searched for components whose abundance was significantly different between cancer ROIs and epithelium ROIs. The analysis was performed either for each tissue specimen separately or for combined ROIs representing all specimens from the training set. Differences between peptide and lipid domains are illustrated below using combined cancer ROIs and epithelium ROIs since pictures obtained for all specimens were similar (see details in the Supplementary Tables S1 and S2). There were 98% of peptide components and 85% of lipid components that showed statistically significant differences in abundance between normal epithelium and cancer (corrected p-value < 0.05). However, classical “p-value statistics” could overestimate the significance of differences between groups with extremely numerous samples (which is typical for MSI, where very large numbers of spectra are registered and compared). Hence, to estimate the significance of differences between compared ROIs the effect size was calculated for each component, which is independent of the number of samples; the Cohen’s d value above 0.5, 0.8 and 1.2 corresponded to medium, large and very large effects, respectively (Cohen 1988). We found that medium, large and very large effect size was observed for 31%, 13%, and 5.7% of peptide components, respectively, and 17%, 9.8%, and 4.3% of lipid components, respectively; the median d-value was 0.49 and 0.31 in the case of peptides and lipids, respectively (Fig. 2c). Hence, there were generally more peptide components than lipid components whose abundances were different between cancer and normal epithelium.

**Fig. 3:**
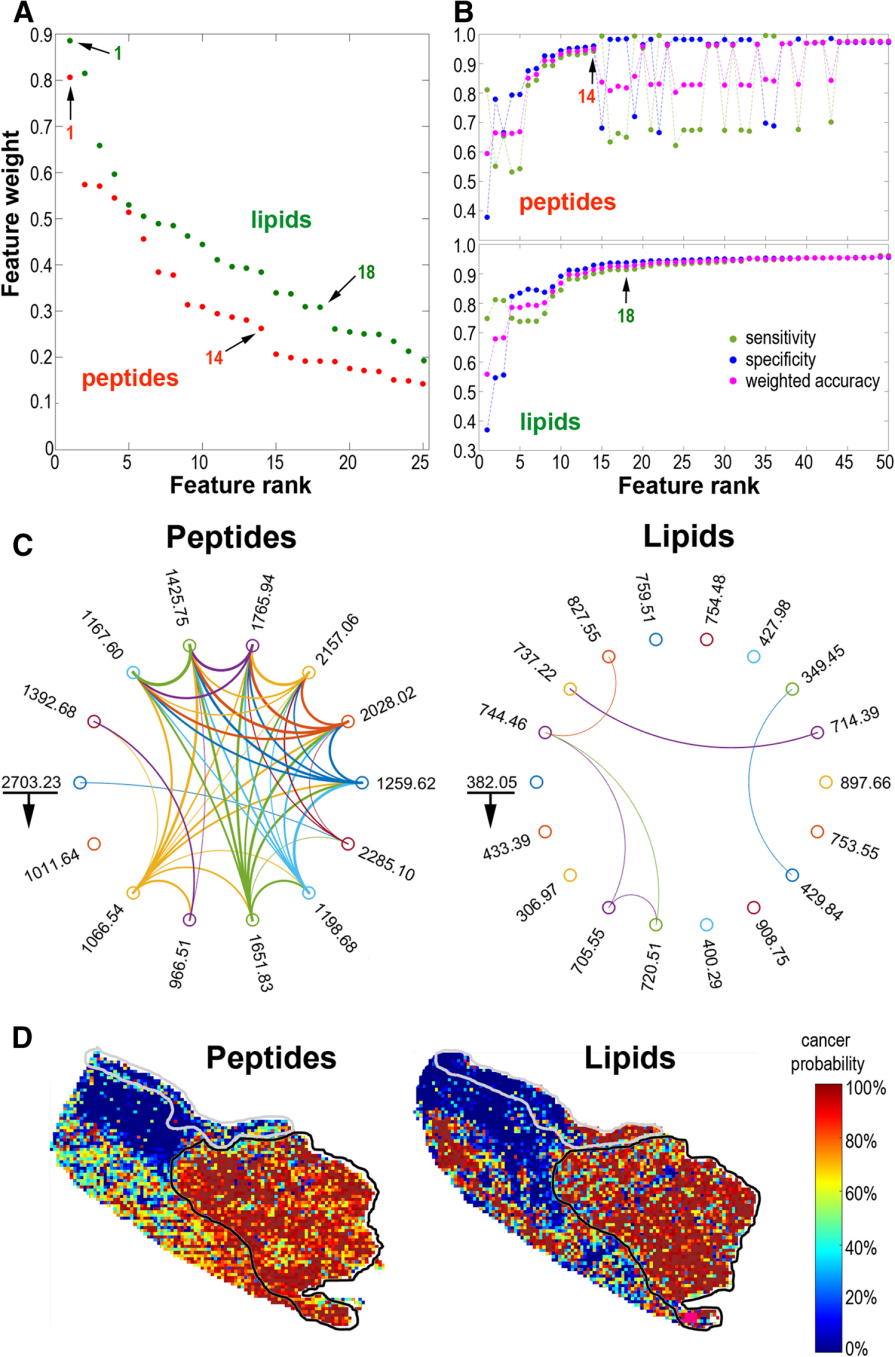
Cancer classifier based on components detected by MALDI–MSI. **a** Rank of top 50 components with decreasing weight in the tested classifiers. **b** Performance of classifiers (sensitivity, specificity, and weighted accuracy) built with panels of features with an increased number of components. **c** Pairwise correlation plot for 14 peptide and 18 lipid components selected for the final classifiers (underlined are top components with the counterclockwise decreasing weight of a component); connected are components of at least high effect size correlation (width of the line represents the strength of the correlation). **d** Results of classification of basic segments (registered spectra) in the validation sample (Case_4); the heat maps illustrate the probability of being classified as < cancer> (grey and black lines delineate expert-determined normal epithelium and cancer, respectively). (Color figure online)

To further compare the ability of peptide and lipid components to discriminate normal and cancerous epithelium cancer classifiers were built and validated using molecular signatures obtained for both domains. Cancer/epithelium classifiers were build using the training set composed of three tissue samples (Case_1, Case_2, and Case_3). The training step allowed to establish the rank of features (i.e., molecular components) that were the most important for classification, and the top features were selected to build a final classifier that was validated using the independent fourth sample (Case_4). Rank of discriminatory components in the tested classifiers is shown in Fig. 3a (see details in the Supplementary Tables S1 and S2). The number of top components selected for the final classifier was based on the knee rule applied to the feature scoring plotted in decreasing order, combined with detection of the plateau (saturation) point for weighted accuracy, classifier sensitivity, and specificity curves (Fig. 3b). There were 14 top peptide features and 18 top lipid features selected for validated cancer/epithelium classifier. pairwise correlation of features from the final classifier signatures was much stronger among peptides than among lipids (Fig. 3c). Both classifiers performed well and allowed classification of registered spectra from cancer ROI and epithelium ROI with very high precision and specificity. This is noteworthy, however, that all indices of cancer/epithelium classifier built on peptide signature were higher than the corresponding indices of a classifier built on lipid signature (Table 2). Moreover, the probability of being classified as “cancer” was estimated for each image pixel (i.e., registered spectrum) from the validation sample Case_4 using the same classifiers. In general, good concordance between the expert knowledge and the results of molecular classification was obtained for both classifiers (Fig. 3d).

**Table 2:** Performance of cancer classifiers built of peptide and lipid components and validated using the independent tissue specimen

Classifier indices	Peptide classifier(14 components) (%)	Lipid classifier(18 components) (%)
Sensitivity	78.7	56.0
Specificity	90.7	82.4
Accuracy	89.5	79.8
Weighted accuracy	84.7	69.2
Precision	97.5	94.4
F-measure	93.9	87.9

The hypothetical identity of MSI components could be established by attributing masses (m/z values) of imaged molecular components to masses of species with a known identity. To look at factors discriminating normal and cancerous epithelium, molecular components with significantly different abundances between cancer and epithelium (d > 1.2) and important for classification of cancer (the top 50) were selected; there were 179 peptide and 124 lipid components that fulfilled either criterion. M/z values of peptide components were attributed to measured masses of peptides identified by the LC–MS/MS in lysates from the same tissue specimens (allowing ± 0.05% mass tolerance limit). Hypothetical identity could be attributed to 147 molecular components detected by MSI (Supplementary Table S3). However, this type of annotation was not unique and more than one identified peptide could be attributed to the majority of MSI components. Nevertheless, proteins whose tryptic fragments were the most frequently attributed to discriminatory MSI components included Neuroblast Differentiation-Associated Protein (AHNAK), Myosin-9 (MYH9), Enolase 1 (ENO1), and Alpha-2-macroglobulin (A2M). This is noteworthy that all these proteins have known cancer-related attributes. Three former ones are highly expressed in several cancer types and may have prognostic value [http://www.proteinatlas.org]. ANHAK is involved in migration and invasion of cancer cells (Sudo et al. 2014), MYH9 could be a tumor suppressor via regulation of p53 (Schramek et al. 2014), while ENO1 is involved in Warburg effect and its targeting sensitizes cancer cells (Capello et al. 2016). Moreover, A2M putatively synthesized by macrophages present in cancer microenvironment could activate cancer cells (Misra and Pizzo 2015). The hypothetical identity of discriminatory lipid components was attributed by annotation of MSI components at the SimLipid database. There were 113 such MSI components with at least one specific compound associated; the most probable hit was attributed to each component based on mass tolerance and expected abundance in a tissue (Supplementary Table S4). There were 56 phosphatidylcholines, 34 sphingolipids, and 16 acylglycerols among compounds hypothetically attributed to lipid components discriminating cancerous and normal epithelium. This is noteworthy that among 18 lipid components comprising cancer classifier there were six species hypothetically attributed to sphingolipids, which could indicate functional importance of this lipid class for cancer-related processes. However, because of the relatively low mass resolution of MALDI–ToF–MSI, hypothetical annotation of components of MSI profiles based on their masses has only limited applicability. Hence, actual identification of peptide and lipid components discriminating normal and cancerous epithelium could be obtained only after on-tissue MS/MS analysis, which was not within the scope of the current work.

## Discussion

We revealed here that a large number of cellular proteins represented by their tryptic peptides imaged by MALDI–MSI showed significantly different abundances between normal and cancerous mucosa. Different proteomics approaches already documented numerous proteins characteristic for HNSCC (Chen et al. 2015; Malik et al. 2016). Therefore, high ability of proteome-based signature to discriminate cancer and normal epithelium could be expected. This is noteworthy, however, that heterogeneity of the proteome profile was much higher within cancerous tissue than within normal mucosa. This apparently indicated intra-tumor heterogeneity and the presence of different cell populations, either actual cancer cells, and cancer-related stroma cells. Direct comparison of protein and lipid domain between paired tissue regions corresponding to oral cancer and normal epithelium was already performed using the Raman spectroscopy. However, due to relatively low spatial and chemical resolution of this approach, the obtained data indicated only increased protein/lipid proportion in cancer regions (Singh and Krishna 2014). MALDI–MSI enabled more specific inspection of a tissue lipidome. In general, enhanced lipid synthesis (lipogenesis) and changes in the composition of membranes (increased contribution of phosphatidylcholines and saturated phospholipids) are typical for cancer phenotype. Furthermore, the increased number of lipid rafts (enriched in cholesterol and sphingolipids) involved in cellular signaling are observed in cancer cells. Moreover, several other lipid components (including free fatty acids, ceramides, and prostaglandins) participate in communication between cancer and stroma cells, which also contributes to cancer-characteristic lipid profile (Santos and Schulze 2012; Beloribi-Djefaflia 2016; Luo et al. 2017). The set of lipid components differentiating cancerous and normal mucosa, which putatively included sphingolipids and phosphatidylcholines, apparently reflected all the above-mentioned processes. Nevertheless, differences between cancerous and normal mucosa were less obvious when corresponding ROIs were compared in respect to the subset of the analyzed lipids. One could assume that the majority of lipid components detected by MSI represented components of cellular membranes. Therefore, the overall similarity of lipid profiles between cancerous and normal epithelium revealed the similar basic composition of lipid membranes in both types of the oral mucosa. Moreover, though variability in lipid distribution was higher within cancer than within normal epithelium, the observed intra-tumor heterogeneity was lower when compared to peptide imaging. However, in spite of the general similarity of lipid composition, at least in respect to the subset of lipids analyzed by positive mode MALDI–MSI, several components with significantly different abundance could be found, which enabled discrimination of cancerous and normal epithelium based on information of lipid distribution. Hence, we concluded that though molecular differences between cancerous and normal mucosa were higher in the proteome domain than in the analyzed lipidome subdomain, imaging of lipidome components also enabled discrimination of oral cancer and normal epithelium. Current proof-of-concept study based on molecular imaging of tissues indicated comparably high feasibility of both proteomic and lipidomic biomarkers of oral cancer. Successively, specific biomarker signatures might be identified and validated by high-throughput approaches using lysates from dissected tissue specimens.

## Electronic supplementary material

Below is the link to the electronic supplementary material.

Supplementary material 1 (XLSX 1509 KB)

Supplementary material 2 (PDF 281 KB)

## Abbreviations

MALDI–MSI: Matrix-assisted laser desorption ionization mass spectrometry imaging
HNSCC: Head and neck squamous cell carcinomas
