# Supplementary material for: Discrimination of normal oral mucosa from oral cancer by mass spectrometry imaging of proteins and lipids
Source: J Mol Histol. 2018 Nov 3;50(1):1–10. doi: 10.1007/s10735-018-9802-3 (PMC6323087; doi:10.1007/s10735-018-9802-3)
Supplement: Supplementary file 2 — Supplementary material 2 (PDF 281 KB) [file 10735_2018_9802_MOESM2_ESM.pdf]

Supplementary material

Discrimination of normal oral mucosa from oral cancer by mass spectrometry imaging of proteins and lipids

Katarzyna Bednarczyk, Marta Gawin, Mykola Chekan, Agata Kurczyk, Grzegorz Mrukwa, Monika Pietrowska, Joanna Polanska, Piotr Widlak

Protocol 1. *Preparation of protein extracts from frozen sections:*

Four sections, 10 µm-thick each, were collected in a 1.5 mL tube for each frozen tissue and centrifuged (675 g, 2 min, 4°C) once thawed. Then, 350 µL of RIPA buffer (1x PBS, 1% NP40, 0.5% sodium deoxycholate, 0.1% SDS) containing proteases inhibitors (Roche) were added to each tube. Tubes were subsequently placed on ice for 20 min and centrifuged (20 000 g, 20 min, 4°C). Each supernatant was transferred to a new tube and subjected to spectrophotometric protein assay according to Bradford method.

Protocol 2. *Tryptic digestion of protein extracts:*

A modified version of the filter-aided sample preparation method coupled with Stage-Tip fractionation was employed for the preparation of extracts for MS-based protein identification. A volume of each extract equivalent to 30 µg of protein was mixed with 8M urea in 0.1 M Tris-HCl pH 8.5 and loaded onto a Microcon YM-30 filter (Millipore) followed by centrifugation (14'000 RCF, 15 min). A portion of 200 µL of urea buffer was added to the filter and centrifuged for another 15 min. A solution of 50 mM iodoacetamide in urea buffer (50 µL) was then added and the filter was left in darkness for 20 min. After that, the filter was centrifuged as above. The filter membrane was subsequently washed three times with 100 µL of urea buffer (each addition followed with centrifugation at 14'000 RCF, 15 min) and another three times with 100 µL of 50 mM Tris-HCl pH 8.5. A solution of trypsin (Sequencing Grade Modified Trypsin, Promega) was prepared in 50 mM Tris-HCl pH 8.5 with an enzyme to protein ratio of 1:100, m/m. A portion of 40 µL of this solution was loaded onto the filter and incubated in a humid chamber for 18h at 37°C. Thus obtained tryptic peptides were recovered from the filter membrane by centrifugation (14'000 RCF, 15 min), followed by washing with water (160 µL H_2O, centrifugation conditions as given above). The obtained portion of tryptic peptides (200 µL) was subsequently subjected to fractionation with the use of Stage-Tips filled with 6 plugs of Empore™ Anion-SR 47 mm Extraction Disk (3M Company, Maplewood, MN, USA): peptides from each sample were separated into two fractions. Elution was performed with 100 µL of 20 mM Britton-Robinson Universal Buffer pH 5 and pH 2. Fractionated peptides were retained on Stage-Tips filled with 3 plugs of Empore™ Octadecyl C18 47 mm Extraction Disk (3M Company) and eluted with 60% ACN 0.1% TFA (50 µL). Extract volume was reduced to one third in a vacuum centrifuge to reduce acetonitrile content.

Protocol 3. *LC-MS/MS analysis of protein extracts:*

Both peptide fractions of each sample (i.e. fraction pH 5 and fraction pH 2) were separated using EASY-nLC (Proxeon) equipped with NS-MP-10 Biosphere column: 100 µm × 2 cm, C18: 5 µm, 120 Å (pre-column) and Acclaim PepMap™ 100 column: 75 µm × 15 cm, C18: 3 µm, 100 Å (analytical column). Buffer A and buffer B consisted of 0.05% TFA/H_2O, and 90% ACN 0.05% TFA, respectively; the mobile phase flow rate was set at 300 nL/min. The total run time was 120 min. Details of the employed gradient elution are given below:

Time, min	Buffer A, %	Buffer B, %
0.00	98.0	2.0
20.00	80.0	20.0
90.00	71.0	29.0
109.00	62.0	38.0
113.34	55.0	45.0
114.00	0.0	100.0
124.00	0.0	100.0
124.10	98.0	2.0
129.00	98.0	2.0

PROTEINEER fc II (Bruker) equipped with an MTP AnchorChip 1536 target plate was used for LC fraction collection. Matrix solution (α-cyano-4-hydroxycinnamic acid) and a solution of external calibrants (Peptide Calibration Standard II, Bruker) were prepared according to *Bruker Daltonics MALDI preparation protocols Life science* (HCCA, dried droplet, nanoLC-MALDI analysis of peptide mixtures). Each sample pH fraction was separated into 680 nano-LC fractions, 10 seconds each (pH fractions originating from the same sample were spotted over the same target plate) with 10 min time delay, and analyzed using ultrafleXtreme MALDI-TOF/TOF mass spectrometer. LC-MALDI measurements were controlled with WARP-LC v. 1.3. software (Bruker). All spectra were acquired in reflectron positive mode. MS spectra were collected within 800-4000 m/z range with matrix deflection up to 700 m/z and PIE of 130 ns, 3000 shots were summed per spectrum at 1 kHz laser repetition rate. Radom walk was activated with 100 shots at raster spot. A maximum number of MS/MS precursors per fraction was set to 10, acquisition order within fraction: acquisition score descending, 4000 shots/spectrum.

Protocol 4. *Protein identification:*

Database search and protein identification were realized with the use of ProteinScape software version 3.1 (Bruker). NCBInr human database was employed with MS tolerance of 100ppm and MS/MS tolerance of 0.4Da; 1 missed cleavage was allowed; Carbamidomethyl (C) and Oxidation (M) were selected as fixed and variable modifications, respectively. Protein list compilation was realized by the search engine (Mascot Server 2.4, Matrix Science, UK). Search engine general settings were as follows: ion score cut-off: 15, min peptide length: 5; protein assessment: accept if Mascot score above 80; peptide assessment: top hit compound only, accept if Mascot score above 20, in case of MS/MS spectra matching peptides from more than one identified protein accept peptide assigned to highest scoring protein.
